# Advancing a cascading train-the-trainer model of frontline HIV service providers in South Africa: protocol of an implementation trial

**DOI:** 10.1186/s13722-021-00236-8

**Published:** 2021-04-30

**Authors:** Caroline C. Kuo, Goodman Sibeko, Morayo Akande, Shaheema Allie, Nurain Tisaker, Dan J. Stein, Sara J. Becker

**Affiliations:** 1 Center for Alcohol and Addiction Studies, Department of Behavioral and Social Sciences, Brown University School of Public Health, P.O. Box G-S121-5, Providence, RI 02912 USA; 2 Department of Psychiatry and Mental Health, University of Cape Town, Cape Town, South Africa

**Keywords:** Alcohol, HIV, South Africa, SBIRT, Implementation

## Abstract

**Background:**

South Africa is marked by high rates of both HIV and alcohol use, and there is a detrimental synergistic relationship between these two epidemics. The Institute of Medicine recommends integrated care for alcohol use treatment and HIV, but implementation of integrated services remains a challenge in South Africa. This protocol describes a study designed to evaluate trainer, provider-, and patient encounter-level outcomes relating to the national rollout of a cascade train-the-trainer model of task-sharing to build capacity of the HIV workforce to deliver Screening, Brief Intervention, and Referral to Treatment (SBIRT) to address risky alcohol use.

**Methods:**

This 5 year protocol consists of two phases. First, we will finalize development of a robust SBIRT train-the-trainer model, which will include an SBIRT Trainer Manual, Provider Resource Guide, fidelity observational coding system, case vignettes, and a curriculum for ongoing consultation sessions. Materials will be designed to build the capacity of novice trainers to train lay workers to deliver SBIRT with fidelity. Second, we will recruit 24–36 trainers and 900 providers in order to evaluate the effects of the SBIRT train-the-trainer model on trainer- (e.g., fidelity, knowledge), provider- (e.g., SBIRT attitudes, confidence, acceptability), and patient encounter- (e.g., proportion receiving screening, brief intervention, referral to treatment) level variables. Data on patient encounters will be tracked by providers on programmed tablets or scannable paper forms in real-time. Providers will report on SBIRT delivery on an ongoing basis over a 6-months period. Additionally, we will test the hypothesis that trainer-level factors will account for a substantial proportion of variability in provider-level factors which will, in turn, account for a substantial proportion of variability in patient encounter-level outcomes.

**Discussion:**

This protocol will allow us to take advantage of a unique national training initiative to gather comprehensive data on multi-level factors associated with the implementation of SBIRT in HIV service settings. In the long-term, this research can help to advance the implementation of integrated alcohol-HIV services, providing lessons that can extend to other low-and-middle income countries confronting dual epidemics.

## Background

South Africa is at the epicenter of the global human immunodeficiency virus (HIV) pandemic, with the largest country population of people living with HIV in the world. An astounding 7.9 million individuals are living with HIV accounting for 14% of the country population [1]. When considering the global burden of HIV, South Africa accounts for 19% of the population living with HIV, 15% of all new HIV infections, and 11% of all deaths related to acquired immunodeficiency syndrome (AIDS) [2]. Currently only 62% of individuals with HIV remain on sustained antiretroviral treatment [1]. This highlights a significant need to improve HIV treatment initiation, adherence, and retention, which in turn will require ambitious efforts to train, mobilize, and build the capacity of the HIV treatment workforce.

South Africa also has high rates of alcohol use and alcohol-related problems, which pose significant challenges to the HIV care cascade. Rates of lifetime alcohol use are 38.7% [3] and alcohol use disorders rank among the three most prevalent lifetime mental disorders in South Africa at 11.4% [4]. Of particular concern, alcohol use is highly prevalent among HIV-positive individuals. In studies of local communities with high HIV prevalence, rates of recent alcohol use are as high as 51% and rates of current alcohol use disorders reach 7% [5]. Moreover, even though rates of alcohol use in South Africa are comparable to other countries, harms experienced per liter of alcohol are much higher in South Africa than in other regions globally [6].

Alcohol use is theorized to exacerbate the South African HIV epidemic via both behavioral and biological pathways, affecting both risk and clinical outcomes. Behaviorally, heavy drinking has been linked to sexual risk behavior and increased HIV infection risk in both cross-sectional and longitudinal studies conducted in Sub-Saharan Africa [7–11]. Meta-analyses show that among people living with HIV, alcohol use is significantly associated with unprotected sex [12], poorer adherence to antiretroviral therapy and lower utilization of health services [13]. Biologically, alcohol use is linked to both increased viral replication and diminished immune function, with a causal effect on the worsening of disease course for HIV [9].

The Institute of Medicine has been recommending integrated care for alcohol use treatment and HIV for over a decade [14]. Benefits of service integration have been noted at the patient-, provider-, and societal-levels, including decreased healthcare costs and improved treatment outcomes [15]. Unfortunately, in South Africa integration of alcohol and HIV service provision has remained a challenge. While South Africa has exceptional HIV treatment expertise, there is a severe human resource shortage for specialist alcohol and other substance use disorder treatment [16]. With only 0.32 psychologists, 0.28 psychiatrists, and 0.4 social workers for every 100,000 individuals, the current workforce lacks the knowledge and skill needed to address the intertwined public health crises of alcohol and HIV [17]. A qualitative study of primary healthcare workers, nurses, and lay workers in South Africa found that health professionals treating HIV lacked basic knowledge about: the criteria for harmful alcohol use; screening tools to assess risky alcohol use; and effective interventions to treat alcohol problems [17]. In recent years, the South African National Department of Health and several national organizations have called for increased training of front-line treatment providers in screening and brief alcohol intervention as a means of mitigating the effects of alcohol on the HIV care continuum [18].

### The South Africa International Technology Transfer Center (TTC)

The South Africa International Technology Transfer Center (TTC) is a national training and technical assistance center formed in October 2020. The core South Africa International TTC infrastructure and team were first formed in 2017 as part of an international network of HIV Addiction TTCs [19] funded by the President’s Emergency Plan for AIDS Relief (PEPFAR) and administrated by the Substance Abuse and Mental Health Services Administration. The overarching objective of the International HIV Addiction TTCs was to build the capacity of the workforce in HIV-endemic countries to address alcohol and other drug use. As is common with PEPFAR funding, a guiding vision was that the International HIV Addiction TTCs would become self-sustaining [20]. This vision was met after three years of PEPFAR funding. The International TTC network was subsequently formed as a program of the International Consortium of Universities for Drug Demand Reduction with initial funding from the U.S. Department of State to serve countries including South Africa, Ukraine, Vietnam, Peru, and Colombia. Although the name and funding has changed, the focus of the International TTCs remains the same. International TTCs are dedicated to building the capacity of the workforce to address alcohol and other drug use, with an emphasis on addressing the “whole patient” and promoting integrated care of alcohol and substance use, HIV, and mental health.

In 2017, as part of the South Africa TTC formation, we conducted a needs assessment [21] of 67 national stakeholders and policy makers to prioritize the areas of greatest training need throughout the country: respondents were from government departments (28.3%), national non-governmental organizations (45.0%), private institutions (20.0%), and community healthcare organizations (3.3%). Seventy percent of the survey respondents employed lay workers (e.g., community health workers, lay counselors), highlighting widespread reliance on treatment professionals without advanced education. By far, the most commonly reported areas of training need were “drug screening” (70%) and “alcohol screening” (60%). Results of this needs assessment were consistent with the recommendations of the South African Medical Research Council that “individual screening for harmful/hazardous alcohol use, brief intervention and referral to treatment (SBIRT) of clients/patients should be performed routinely by trained health care workers using standardized treatment protocols and screening tools [18].” Thus, the South Africa International TTC made the strategic decision to select Screening, Brief Intervention, and Referral to Treatment (SBIRT) focused on risky alcohol use as a priority intervention for national roll-out.

### SBIRT

SBIRT is an evidence-based, cost-effective approach to identifying people with alcohol use disorders and those at risk of developing these disorders, in order to deliver early intervention and treatment to people who may benefit [22–24]. Screening is used to quickly assess the severity of alcohol use and identify the appropriate level of intervention. When deemed necessary, a brief intervention then focuses on increasing awareness regarding the risks of alcohol use as well as building motivation toward making a behavioral change. Those identified as needing more extensive treatment with access to specialty care are provided with a referral to treatment. There is robust evidence in support of SBIRT in South Africa [25, 26] and several implementation initiatives have demonstrated that it is feasible to train a range of health professionals to deliver SBIRT [27]. Key questions remain, however, about whether it is feasible and effective to train health professionals and lay workers to implement SBIRT in low-resourced HIV care settings like South Africa. The current protocol aims to address this gap in our knowledge by conducting a multi-level evaluation (outcomes evaluated at the trainer-level, provider-level, and patient encounter-level) of a comprehensive train-the-trainer model as a strategy to advance the implementation of SBIRT in HIV care settings throughout South Africa.

### Advancing implementation science for integrated alcohol-HIV treatment

Implementation science has been defined as the “the scientific study of methods to promote the systematic uptake of research findings and other evidence-based practices into routine practice, and hence, to improve the quality and effectiveness of health services [28].” Central to the South Africa International TTC’s implementation science approach is the strategy of task sharing to meet severe shortages in specialist practitioners who can provide services to those in need. Task sharing, a strategy recommended by the World Health Organization, involves the re-distribution of treatment tasks to non-specialists with less skills and experience with health interventions, who in turn receive training and supervision from specialists [29, 30]. The ultimate goal is to increase the capacity of health systems by improving prevention and treatment access. The South Africa International TTC has developed a model of cascading training (i.e., train-the-trainer model), which represents a vital component of a broader task sharing approach. In the HIV field, studies of task sharing show that lay workers without specialized training can be trained to deliver effective health interventions in South Africa and other low- and middle-income countries [31, 32]. By contrast, studies of task sharing have not yet focused on the delivery of substance use disorder treatment, which limits the ability to bring integrated alcohol-HIV care to scale. Another key issue that has yet to be explored in the task sharing literature is whether train-the-trainer models, in which a master trainer provides didactic content to novice trainers, who in turn train other health professionals and lay workers, are effective in building the capacity of the workforce to implement evidence-based interventions with fidelity. Answering this question requires comprehensive assessment of implementation outcomes [33], which are fundamentally distinct from traditional clinical outcomes. Moreover, implementation outcomes must be assessed at multiple levels including the trainer-, the service provider (e.g., health professional or lay worker), and the patient encounter-levels.

### Pilot data on SBIRT implementation in South Africa

Preliminary evidence for the promise of implementing SBIRT in South Africa comes from a small pilot study of SBIRT training conducted by the South Africa HIV Addiction TTC [34]. We asked providers from the Kwazulu-Natal Department of Health, a provincial Department of Health in one of the PEPFAR priority regions, to report on their rates of screening, brief intervention, and referral to treatment delivery in the 3 months prior SBIRT training received from the South Africa HIV Addiction TTC, as well as in the 3 months after this SBIRT training. A total of 38 providers completed a brief self-report tool. Based on provider report, rates of patient screening increased from 38 to 50% pre- and post-training; rates of delivery of brief intervention increased from 22 to 47%; and rates of referral to treatment remained fairly steady from 15 to 18% [34].

Additionally, qualitative feedback from the providers about how their receipt of SBIRT training affected their practice revealed a number of encouraging themes. These included increased recognition of the need to address alcohol misuse within HIV care; increased comfort discussing alcohol misuse with HIV patients; increased comfort providing a brief intervention; and beliefs that patient outcomes were improved due to training. As an example, one HIV treatment provider said, *“There was this client who had a problem with alcohol use. After we did the brief intervention she decided to reduce her drinking because her viral load was off the charts. [It is] now low[er] than detectable as she is taking treatment accordingly.”* The qualitative results, as well as the quantitative trends described above, are promising and provide preliminary support for the objectives of the present protocol [34].

### Study objectives and specific aims

**Fig. 1:**
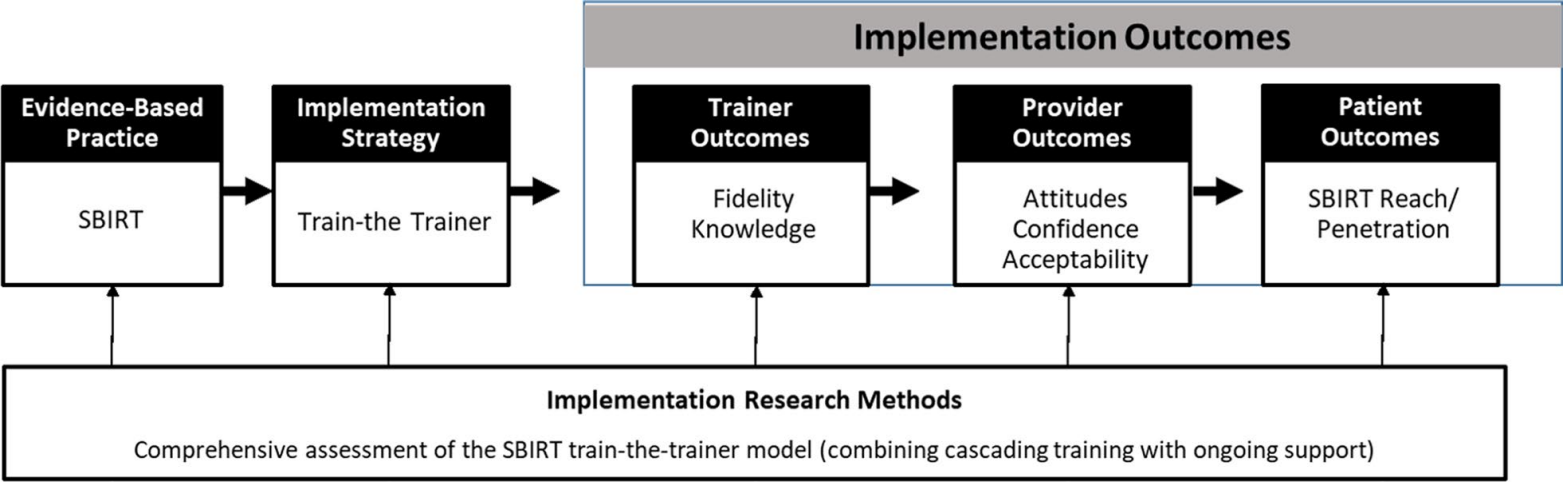
Conceptual model of the proposed implementation research

The overarching goal of this protocol is to advance our knowledge of the influence of a cascading train-the-trainer model as a means of promoting the uptake of SBIRT by a broad range of service providers, including both health professionals and lay workers. We will measure the impact of a train-the-trainer model in a country at the epicenter of the alcohol and HIV epidemics. One notable innovation of this protocol is our planned focus on task sharing to advance the uptake of SBIRT. Task-sharing has been successfully used in HIV care [35, 36], and despite its promise for addressing barriers to substance use treatment, it has only recently been applied to alcohol interventions. A second innovation involves development of a measure of trainer fidelity, which captures both trainer adherence and competence.. To date, implementation science efforts have yielded a plethora of provider fidelity scales [37, 38], but a dearth of trainer fidelity measures, which are essential to the task-sharing model. We will conduct a comprehensive evaluation of the South Africa International TTC’s SBIRT cascade training initiative by gathering outcome data across multiple levels (i.e. trainer, provider, and patient encounter), which in turn can inform a robust approach to increasing the scalability of integrated alcohol-HIV care in low-and-middle income countries. Consistent with the Conceptual Model of Implementation Research developed by Proctor and colleagues [39], Fig. 1 presents an overview of the specific intervention strategy, implementation strategy, and multi-level outcomes that will be used in this protocol.

#### Specific aim 1

To finalize an SBIRT train-the-trainer model—consisting of an SBIRT Training Manual, Provider Resource Guide, case vignettes, fidelity monitoring coding system, and a protocol for provision of ongoing consultation—to promote SBIRT scalability to health professionals and non-specialist health workers.

#### Specific aim 2

To evaluate the effectiveness of the SBIRT train-the-trainer model on key implementation outcomes measured at the trainer- (i.e., knowledge and fidelity), provider- (i.e., confidence, attitudes, and acceptability), and patient encounter- (i.e., proportion of sessions in which patients are screened and receive a brief intervention) levels.

#### Specific aim 3 (Exploratory)

To explore the extent to which the multi-level variables interact. Specifically, we examine the extent to which outcomes measured at the trainer-level predict outcomes measured at the provider-level, and in turn, the degree to which variables measured at the provider-level predict outcomes measured at the patient-encounter level.

## Methods

### Phase I procedures

Phase 1 (Aim 1) will involve development of the SBIRT train-the-trainer resources. As outlined below, this includes development of the (1) train-the-trainer curriculum, (2) trainer fidelity checklist, and (3) protocol for ongoing provider support.

#### Train-the-trainer curriculum development processes

The South Africa International TTC will develop a detailed train-the-trainer curriculum, which will be built around screening tools with psychometric support in South Africa and a set of case vignette training aides. The South Africa International TTC currently has an SBIRT training curriculum, which is appropriate for expert trainers to train seasoned health professionals. Such content will be substantially adapted to ensure that it is appropriate for health professionals (with limited experience delivering training) to train lay workers (with minimal pre-existing skill in alcohol or alcohol-relevant interventions).

#### Screening tool selection

The South Africa International TTC train-the-trainer curriculum will center around the Alcohol Use Disorders Identification Test (AUDIT), a 10-item questionnaire that was developed using data from a World Health Organization multi-national collaborative study, as the primary tool [40]. The AUDIT will be used as a focal screening tool because it is the only publicly available questionnaire that has been developed specifically for international use, has been translated into over 20 languages, and has demonstrated strong psychometric properties in South African treatment settings [41]. Scores for each item range from 0 to 4 with 4 denoting the highest level of risk: total scores range from 0 to 40. AUDIT scores will be used as part of a risk algorithm to tailor alcohol use intervention services to each individual’s risk profile. The SBIRT training curriculum will use the risk categories shown in Table 1, which align with recommendations of the South African Medical Research Council and the World Health Organization [42]. Instruction in this risk algorithm will comprise a major portion of the SBIRT training curriculum.

**Table 1:** AUDIT-based risk algorithm and intervention recommendation

Zone	Score Range	Recommendation
1: Low risk	0–7 (no or infrequent hazardous drinking)	Positive feedback paired with simple information about alcohol and its effects on HIV
2: Medium-risk	8–15 (some hazardous drinking)	Brief intervention (e.g., brief risk reduction conversation based on Motivational Interviewing)
3: High-risk	16–19 (high-risk drinking causing harm)	Brief intervention paired with active case management
4: Very high risk	20–40 (probable alcohol use disorder)	Brief intervention paired with referral to more intensive treatment for an alcohol use disorder to reduce risk of re-infection, treatment non-adherence, disease progression, and serious alcohol-related consequences

#### SBIRT trainer manual and provider resource guide development

The SBIRT Trainer Manual will begin with a robust set of materials to support the delivery of didactic training by novice trainers to health professionals and lay workers. Didactic materials will cover three key themes. First, materials will highlight the latest evidence around effective SBIRT delivery. Evidence in support of SBIRT will be conveyed at a secondary school level using simple language, visual pictures, and figures. This scaling of materials to a secondary school level is critical because many lay workers may have been denied access to education during the Apartheid regime but are capable of quality SBIRT delivery [43, 44].

Second, the Trainer Manual will provide detailed instruction in the skills needed to deliver training effectively. General skills needed for SBIRT delivery, which will be included in the manual and covered in the South Africa International TTC-led train-the-trainer sessions, will include: (a) use of role-play to develop skills of lay workers; (b) ways to actively listen and draw out discussion from providers to promote engagement; (c) strategies to solicit and troubleshoot barriers to SBIRT implementation in specific contexts; d) activities to make learning around SBIRT delivery interactive (i.e., ice breaker activities, vignettes); (e) time management strategies; and (f) ways for trainers to gauge how well trainees are taking on skills. In addition, specific skills needed to address local contextual factors will be covered. For instance, the Trainer Manual will address strategies to deliver training in rural areas that often lack basic amenities (e.g., use of handouts instead of slides in case the site lacks electricity; use of downloaded videos instead of website-hosted videos in case site lacks internet) as well as techniques to ensure materials are conveyed at a secondary school level.

Finally, the Trainer Manual will provide all of the materials and checklists needed to facilitate training (e.g., handouts, didactic slides, mock-ups of the images and text that would need to appear on flipcharts, case vignettes to guide role-play, ice-breaker activities, etc.). The Trainer Manual will also contain detailed instruction in how to administer pre- and post-training knowledge tests as a means of adjusting training content to the level of attendees, as well as providing immediate corrective feedback to those training attendees that do not demonstrate proficiency with concepts at the culmination of training.

The trainer resources will be augmented by a Provider Resource Guide, which will be given to individuals receiving SBIRT training and will contain step-by-step instructions of SBIRT delivery along with detailed instructions in AUDIT administration and scoring. The Participant Resource Guide will contain images of the typical SBIRT workflow and links to video vignettes of effective SBIRT delivery (described below).

#### Case vignette development

In order to enhance widespread scalability, the South Africa International TTC will develop a set of video and text-based exemplar case vignettes. Videos will demonstrate both effective and ineffective administration of the AUDIT, with a focus on how to administer AUDIT items, how to use the AUDIT to determine a patient’s risk level, and what steps to take following AUDIT scoring. Locally relevant video vignettes will also demonstrate how to conduct a brief intervention using principles of motivational interviewing. Videos will be posted publicly on the South Africa International TTC website. A set of preliminary videos, which will be carefully reviewed and expanded to provide additional examples, can be viewed at the following link: https://attcnetwork.org/centers/south-africa-hiv-attc/free-training-videos [45]. Videos will be augmented by a set of text-based standardized case vignettes, which providers can use to role play SBIRT administration during the training sessions.

#### Trainer fidelity coding system development

Following the finalization of a Trainer Manual and accompanying resources, we will develop a fidelity monitoring observational coding system designed to capture the core ingredients needed for training skills in SBIRT. The goal of this fidelity monitoring coding system is to ensure a process for capturing the quality and consistency of the delivery of SBIRT training skills by trainers to health professionals and lay workers.

The coding system will contain elements of both trainer adherence (i.e., the consistency with which they delivered SBIRT training components) and trainer competence (i.e., the skill with which they delivered SBIRT training components), consistent with recommendations by Carroll and colleagues [46]. In the adherence dimension, the system will focus on coding elements such as coverage of key SBIRT components and inclusion of specific training components including didactic information, role-plays, and experiential learning. Such items will be coded fully/partially/not at all to denote whether the component was covered. In the skills dimension, the system will focus on rating the extent to which the trainers demonstrated effective skills such as participant engagement, time management, and responsiveness to questions. Consistent with other well-established scales of provider competence [47, 48], items will be coded on a 1–5 scale with 1 indicating “absent” and 5 indicating “fully present.” A coding manual will be provided to support reliable coding by an independent member of the South Africa International TTC who has knowledge of the SBIRT curriculum but who was not involved in training delivery. A score of 3 or above on each item will indicate an acceptable level of skill in training delivery.

#### Development of protocol for ongoing provider consultation

In addition to developing a Trainer Manual and fidelity coding system, our task sharing model will include the development of curriculum for ongoing consultation sessions. Consistent with the growing recognition that didactic training is more effective when paired with ongoing consultation [49, 50], all health professionals and lay workers that receive SBIRT training will have the opportunity to join monthly videoconference consultation calls, led by SBIRT trainers and coordinated by the South Africa International TTC. Each consultation call will begin with a brief didactic lesson about SBIRT delivery (approximately 15 min), and then the remainder of the session will be dedicated to a case presentation (i.e., example of HIV patient that receives screening for risky alcohol use that merits some form of brief intervention) followed by interactive group discussion. The South Africa International TTC team will develop a set of 12 brief didactic lessons covering themes such as: integrating HIV risk assessment into SBIRT delivery; detecting inconsistent screening responses; building motivation via the brief intervention; and effective referral to treatment.

In harmony with a task sharing approach, SBIRT trainers who completed the train-the-trainer session will be responsible for leading the didactic portion and soliciting volunteers for the case presentations. The goal of the consultation calls is to form a virtual hub-and-spoke knowledge sharing network, with the SBIRT trainers as the “hub” and the health professionals and lay workers as the “spokes” delivering SBIRT nationwide [51]. Each provider attending SBIRT training will be welcome to participate in monthly consultation calls for up to one year. The goal of these multi-point videoconference calls is to enhance the sustainability of the SBIRT training by reinforcing SBIRT skills on a routine basis.

#### Material refinement based on stakeholder feedback

Once the train-the-trainer curricula components have all been drafted (including the Trainer Manual, fidelity coding system, and ongoing consultation call curricula), the materials will be reviewed by members of the South Africa International TTC National Advisory Board. The National Advisory Board is comprised of over 30 representatives from 20 different national agencies and organizations^1^ and convenes twice per year to provide expert guidance to the South Africa International TTC. We will share the draft Training Manual and solicit feedback on the following themes, designed to help us refine and finalize the manual for roll-out in the field: a) ease and clarity of use by trainers; b) whether the manual captures the context and culturally specific elements needed for use in South Africa; c) whether examples used in the manual and learning exercises such as role-plays allow context-specific nuances across different cultural and language groups to surface adequately; d) whether core components of SBIRT are adequately covered; and e) whether skills needed for effective delivery of training to providers by trainers have been covered in sufficient depth.

### Phase 2 procedures

Phase 2 will involve implementation and evaluation of the SBIRT train-the trainer model described above, in order to address Study Aims 2 and 3. We will examine implementation of SBIRT using trainer-, provider-, and patient encounter-level outcomes over a 3-year horizon. Data will be collected immediately prior to the training, immediately after training, and again at 3- and 6-month follow-up assessments.

#### Participants

Participants will include a total of 24–36 trainers and 900 providers embedded within three distinct organizations (8–12trainers and 300 providers per organization). We will partner with three organizations or departments responsible for provision of HIV services, each of which has broad reach in HIV endemic regions and has a productive relationship with the South Africa International TTC. To be eligible for this protocol, partners must provide healthcare services to individuals in key populations either diagnosed with HIV or at heightened risk of HIV-related consequences (e.g., sex workers, inmates in correctional facilities, adolescent girls and young women). Each partner organization will nominate staff to receive SBIRT instruction from the South Africa International TTC and to become certified SBIRT trainers. To be selected as an SBIRT trainer, individuals must meet the following criteria: (a) be currently registered with a regulatory body relevant to HIV care (e.g., registered counselor, nurse, medical doctor, psychologist, social worker); (b) have been with their current organization for at least 1 year; and (c) have experience providing training and/or supervision to direct front-line treatment providers. Following nomination by their organization, the South Africa International TTC team will conduct a brief screen to confirm eligibility.

At each organization, the 8–12 SBIRT trainers will in turn roll-out the curriculum to a minimum of 300 providers (average training size = 25–30 participants), thereby reaching approximately 900 providers in Years 2–4 (300 providers per organization * 3 organizations = 900 providers). Providers selected to receive the training will include the full range of treatment providers whom our partner organizations seek to train in effective alcohol screening and brief intervention. Based on our initial needs assessment, we expect our training attendees to be predominantly (approximately 70%) lay workers, with some representation of registered counselors, nurses, doctors, psychologists, and social workers. We also expect the demographics of the behavioral health workforce (both trainers and providers) to generally mirror the composition of the South African population, as reflected in the most recent census: Black African (80.2%), White (8.4%), Coloured—a South African census term for a multiracial ethnic group—(8.8%), Indian (2.5%), and Other/Unspecified (0.5%; [52]).

#### Train-the-trainer model delivery

##### International TTC led train-the-trainer sessions

The South Africa International TTC will deliver SBIRT train-the-trainer sessions to those individuals nominated to become SBIRT trainers. These TTC-led sessions will be 2-days in duration, with assessments collected both pre- and post-training. The focus of these sessions will be to provide instruction in how to train in SBIRT. Attendees will receive the Trainer Manual developed in Phase 1, which will have all of the resources needed to deliver training to providers with limited experience delivering alcohol interventions (e.g., health professionals and lay workers).

Train-the-trainer sessions will culminate in substantial time spent practicing training delivery, with attendees pairing up to “role play”: one individual will practice the role of SBIRT trainer and the other will practice the role of training attendee. These role plays will be observed by a South Africa International TTC trainer, who will use the fidelity coding system to provide immediate feedback. If any of the trainers do not meet the pre-defined benchmark (see Measures) in their role play, the South Africa International TTC trainer will offer feedback on areas in need of improvement. For those trainers who meet the benchmark, positive reinforcement will be provided.

All attendees must complete a post-training knowledge test and at least one role play that meets the benchmark prior to being deemed a certified SBIRT trainer (see Measures for description of scoring and benchmarks). A specific criterion score on the post-training knowledge test is not required to proceed; however, those attendees scoring below the target will receive detailed corrective feedback from a South Africa International TTC trainer to ensure that they understand the key concepts covered in the training. By contrast, attainment of the benchmark score on at least one role play will be required. Those attendees who are unable to meet this requirement by the culmination of the 2-day session will be scheduled for Zoom practice sessions with the South Africa International TTC team. If a trainer does not meet the benchmark after three consecutive attempts, the partner organization will be notified and will have the option of sending the trainer back for re-training or selecting an alternate trainer. Once the benchmark is met, the attendees will be deemed official International TTC-certified SBIRT trainers.

##### Training sessions led by new SBIRT trainers

The certified SBIRT trainers will be required to lead at least one training within 3-months of their completion of the International TTC instruction. Each training will be 20–30 attendees, each of whom will receive the Provider Resource Guide. These SBIRT training sessions will be 1-day in duration. Training sessions will follow the same curriculum as the SBIRT train-the-trainer sessions, but will be streamlined given the removal of the content on how to effectively lead a training session. As with the initial train-the-trainer curriculum, there will be a substantial emphasis on role plays, though in these training sessions the role plays will focus on SBIRT delivery. Attendees will again pair up with one attendee playing the role of provider and another attendee playing the role of patient, using standardized case vignettes included in the Provider Resource Guide. Those providers receiving SBIRT training will be followed for 6-months and will provide outcome data at four distinct timepoints: immediately prior to didactic training, immediately post-training, 3-months post-training, and 6-months post-training, as elaborated in the following sections.

##### Ongoing monitoring and consultation

Ongoing support will be offered for both the SBIRT trainers and the providers in the form of periodic live observation and ongoing consultation calls. For certified SBIRT trainers, the South Africa International TTC team will go out into the field and live observe all of the scheduled SBIRT training sessions. The trainers will be informed in advance that their sessions will be observed as part of the ongoing monitoring process. Using the same protocol as the role play sessions, the observer will live code the training sessions using the fidelity coding system. The observer will provide private feedback at the end of these training sessions on training delivery in terms of both adherence and skill.

For providers, the South Africa International TTC will hold hourly videoconference consultation calls the last week of each month every day of the work week (i.e., five calls per month). The agenda and curriculum for these calls was described in Phase 1. Because SBIRT training rollout will be staggered and calls will be optional, attendance is expected to be manageable. More frequent sessions will be offered if attendance consistently exceeds more than 25 training recipients per call. Both attendance and engagement in these calls will be tracked as indicators of engagement in the train-the-trainer curriculum.

##### Post-training data capture

To evaluate the impact of training, attendees of the SBIRT training will commence recording data on their delivery of the AUDIT during patient encounters immediately after training with continuous recording of data for 6 months. As elaborated in the Measures section, patient encounter data will be tracked via programmed tablets or scannable paper forms, which will depict the AUDIT items and scoring algorithm.

Tablets and high-speed scanners will be offered to the partner organizations as an incentive for study participation: incentives are offered at the organization-level, reflecting the organization-wide focus of the train-the-trainer strategy. Each tablet and scanner will come with a brief user guide with step-by-step images showing how to answer the AUDIT questions using the technology. Tablets will enable off-line data collection and automated scoring using a highly intuitive user interface and robust data capture system, based around a platform called Survey2Go, though eventual connection with internet will be needed to submit data to the research team.

Our research team has developed and tested pre-programmed tablets as assessment administration and data collection tools with health professionals and lay workers in prior studies in South Africa [53]; the system has been applied in a wide range of contexts in South Africa ranging from no- to low-bandwidth, intermittent electricity, and in community and clinical contexts in both urban and rural areas. When tablets are not available or pragmatic such as in remote areas where even periodic internet connection is unlikely, providers will administer the AUDIT on user-friendly, one-page scannable forms using paper and pencil. Scannable forms will be sent to the research team in weekly batches for cleaning and aggregation.

### Measures

Prior to the start of training activities, leaders at each partner organization will fill out an Organizational Background Form that will contain data about key contextual factors that might be relevant to implementation including provinces served, key populations served, patient census, number of providers employed, and specific HIV services provided.

Once training activities commence, data will be collected from trainers, providers, and from case report forms capturing patient encounter data. The timeline of measure administration for the trainer-, provider-, and patient encounter-level data is shown in Table 2. For both trainers and providers, additional data collection will include basic socio-demographics including years of experience in the field, years of employment at the organization, biological sex, gender, South African population group (e.g., the equivalent of race/ethnicity in the United States), and education.

**Table 2:**
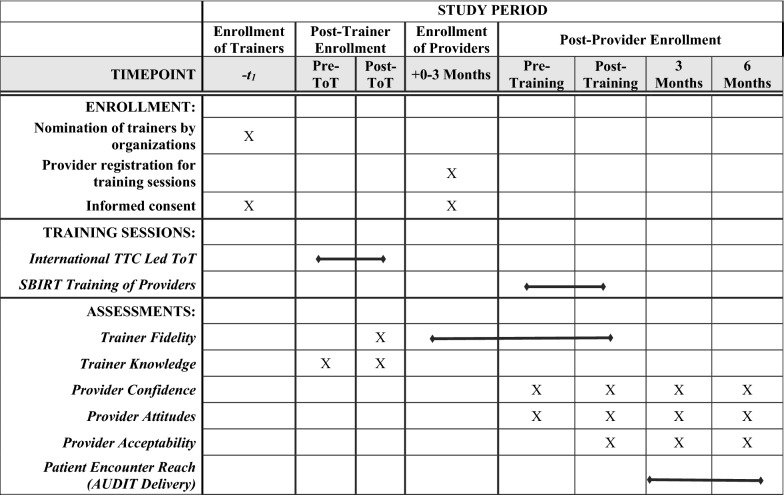
Schedule of enrollment, training sessions, and assessments

*ToT* train-the-trainer; *TTC* Technology Transfer Center

#### Trainer-level measures

##### Trainer knowledge

At both the start and the end of South Africa International TTC-led training sessions, the attendees (the “SBIRT trainers”) will complete an SBIRT Knowledge Measure. This 20-item scale is based on an SBIRT Knowledge Test previously used in an SBIRT training initiative funded by Knopf-Amelung and colleagues [54], and has been tailored for the South African context. The scale contains true/false and multiple-choice items that assess knowledge of screening (e.g., how AUDIT scores are matched to risk zones) and brief intervention (e.g., steps of a brief interview, examples of motivational interviewing skills). A proportion of the number of correct items is used. Absolute scores range from 0 to 20, with 20 denoting the highest level of knowledge: these scores will be changed to proportions ranging from 0 to 100%. Attendees scoring below 70% on the post-training knowledge assessment will receive performance feedback from a member of the South Africa ITTC team.

##### Trainer fidelity

As noted previously, at the end of the International TTC led sessions, the International TTC team will observe the attendees completing role plays of training delivery. In addition, SBIRT training sessions will be observed to monitor fidelity. The South Africa International TTC observer will live code the trainer’s adherence and skill using the SBIRT Training fidelity monitoring checklist described in the “Trainer Fidelity Coding System Development” section. To score adherence, a point will be given for each training element fully covered and a half point will be given for each element partially covered. To score competence, each skill item will be rated from 1 to 5. The fidelity targets for both role plays and live observation will be 70% adherence, and a score of 3 or above on each of the skills items (i.e., “adequate” competence).

#### Provider-level measures

##### Provider attitudes

At four timepoints (pre-training, post-training, 3-months post-training, and 6-months post-training), providers will answer 10 items that capture both positive and negative (reverse scored) attitudes towards SBIRT. Example items include “Clients will be angry if I ask these questions” and “My involvement with a client can make a difference regarding his/her alcohol use.” Items are scored on 5-point scales from “Strongly Disagree” to “Strongly Agree.” Possible scores range from 10 to 50, with 50 denoting the best possible attitudes towards SBIRT. Measures of provider attitudes are from the SBIRT Implementation Toolkit developed by the Emergency Nurses Association [55].

##### Provider confidence

At the same time intervals as the provider attitudes scale, providers will answer 7 items that capture providers’ confidence in their ability to deliver SBIRT. Each item begins “I am confident in my ability to.” Example stems include “ask patients about their alcohol use,” “refer patients with alcohol problems,” and “discuss/advise patients to change their drinking behavior.” Items are scored on 5-point scales from “Strongly Disagree” to “Strongly Agree.” Possible scores range from 7 to 35, with 35 denoting the highest level of confidence. This scale is also from the SBIRT Implementation Toolkit [55].

##### Provider acceptability, appropriateness, and feasibility

Three brief, 4-item measures of acceptability, appropriateness, and feasibility will be administered at the same time intervals as the aforementioned provider scales. This collection of measures was developed by Weiner and colleagues [56] to facilitate pragmatic assessment of leading indicators of implementation success: the measures can be used separately or together and are designed to allow customization to a specific evidence-based intervention strategy. Acceptability will be measured via the Acceptability of Intervention Measure, which will contain the items “SBIRT is appealing to me” and “SBIRT meets my approval.” Appropriateness will be measured via the Intervention Appropriateness Measure, which will have items including “SBIRT seems applicable” and “SBIRT seems like a good match.” Feasibility will be measured via the Feasibility of Intervention Measure, which will have items “SBIRT seems implementable” and “SBIRT seems doable.”

#### Patient encounter-level measures

To evaluate how SBIRT training impacts provider-patient encounters, we will measure data from all patient encounters in the participating units of organizations in real-time using brief session report forms. These forms will be collected via programmed tablets and/or scannable forms administered by providers to patients. Patient encounter-level data will include the patient’s gender, race, HIV status, and whether the patient is currently receiving HIV treatment.

Additional fields will include documentation of the AUDIT screening results. The first two fields will be used to indicate (1) whether or not the patient is screened for alcohol use (yes/no) and (2) scores for each of the 10 items on the AUDIT. When completed via tablet, the sum of AUDIT items contained in Question 2 will auto-calculate. When completed via paper, providers will be given a basic scoring template to calculate and interpret the score. A user-friendly table will be provided that summarizes the patient’s risk level and recommended intervention in accordance with the risk algorithm(modeled after Table 1), while the tablet will directly indicate which level of care is recommended. This will result in field (3) recommended action based on AUDIT (which will either be automated on the tablet or clearly noted on the paper forms based on score), and then the provider can enter field (4) final action taken (no action taken, positive feedback / encouragement about drinking given, brief intervention provided, or brief intervention provided with referral to specialty care). Data collected using these fields will be used to assess a patient encounter-level measure of reach, calculated as the proportion of total patient encounters over the specified recall time period during which the patient received screening, brief intervention, and referral to treatment.

## Analysis plan

Aim 1 (creation of a Trainer Manual) will not require formal data analysis. For Aim 2, all SBIRT trainers (n = 24–36) and all providers who receive SBIRT training (minimum n = 900) will be included in the statistical analyses [57, 58]. Prior to the main analyses, we will run descriptive statistics on the outcomes collected as part of Aim 2 to examine distributional properties, identify outliers, and transform variables as needed. Missing data will be imputed following recommendations of the National Research Council [59]. We will test the appropriateness of assumptions underlying the use of multiple imputation using sensitivity analyses [60].

For Aim 2 evaluation of trainer outcomes, the proportion of training sessions that met the fidelity benchmark will be examined as an outcome of interest, with the goal that at least 80% will meet the target. In addition, we will examine pre-training to post-training change in trainer SBIRT knowledge and proportion of trainers who attained the 70% knowledge benchmark immediately at the culmination of training.

For Aim 2 evaluation of provider- and patient encounter-level variables, change in each outcome will be examined over three distinct time intervals: 3-months pre-training to training (i.e., baseline reports of the past 90 days); training to 3-months post-training; and 3- to 6-months post-training. We will use longitudinal models to examine whether the provider- and patient encounter-level implementation outcomes significantly change across 3 assessments. Repeated measures and nested structures (due to providers being nested within trainers) will be accounted for by adding appropriate random effect terms. The longitudinal models will be estimated using generalized estimation equations which lead to robust standard error and statistical inferences [61]. Using the models, we will examine the outcomes of each post-training visit and compare them with the pre-training outcome. An omnibus test also will be conducted to compare all post-training visit outcomes with the pre-training outcomes. In addition to reporting statistical significance, we will report the effect size using Cohen’s d [62].

For Aim 3 exploratory analyses, we will test a series of multilevel models in which patient encounters (Level 1) are nested in providers (Level 2) which are nested within trainers (Level 3). Consistent with Raudenbush and Bryk’s multilevel modeling strategy, the proportion of variance to be explained at each level will be examined as an initial step [63]. Next, following a decomposed-first strategy that advocates for starting with moderation-focused hypotheses to avoid biases associated with conflated effects, we will conduct multilevel regressions testing the extent to which any putative covariates significantly moderate the hypothesized relationship among the variables [64]. We will test putative covariates (e.g., years of experience, biological sex, population group) at two levels: trainer and provider. All tests of moderation will adjust for the multi-level nature of the nested variables. If moderation is not found (e.g., if biological sex of the trainer does not moderate the effect of fidelity on provider attitudes), covariates will be controlled for as predictors. The goal of these analyses will be to determine the proportion of variance in patient encounter-level outcomes accounted for provider-level outcomes, and the proportion of variance in provider-level outcomes accounted for by trainer-level outcomes, after controlling for putative covariates. These data will help to elucidate mechanisms of action underlying the train-the-trainer cascading model.

### Power analysis and sample size

We used data from our preliminary pilot work to formulate a realistic range of necessary assumptions for sample size and power considerations for primary outcomes (Table 3). Using our most conservative assumptions that rates of referral to treatment will only increase by 5% (at the lower end of rates reported in our preliminary study) and that the repeated measure correlation is only 0.3, we would need a complete sample of 488 to have sufficient power achieve power of 0.80 with a two-tailed alpha of 0.05. We expect an attrition rate of 20% over time (our prior studies in South Africa have attained retention rates of 85–100%): if we were to conservatively assume an attrition rate twice as high as expected (40%), we would need a sample of 683 to have adequate power. The proposed sample size of 900 is therefore extremely well powered to detect change in rates of screening, brief intervention, and referral to treatment over the course of the study. We propose 900 because it will increase our power to detect significant moderators: power will vary based upon the specific hypothesis and will be calculated using PowerUp! Software [65], but should be adequate to detect moderator effects at the provider- and provider encounter-level data. In addition, our proposed sample is reflective of the South Africa International TTC’s intention to roll out SBIRT nationally and train large cadres of health professionals and lay workers.

**Table 3:** Power analysis and sample size (power = .80, alpha = .05)

	Repeated measure correlation	Pre-training rate	Post-training rate	△	Sample Size	
Screening	0.3	25%	40%	15%	107	
		25%	50%	25%	41	
		25%	60%	35%	22	
	0.5	25%	40%	15%	77	
		25%	50%	25%	30	
		25%	60%	35%	16	
Brief Intervention	0.3	20%	40%	20%	58	
		20%	50%	30%	28	
		20%	60%	40%	16	
	0.5	20%	40%	20%	43	
		20%	50%	30%	21	
		20%	60%	40%	13	
Referral to Treatment	0.3	10%	15%	5%	488	
		10%	20%	10%	142	
		10%	25%	15%	71	
	0.5	10%	15%	5%	353	
		10%	20%	10%	104	
		10%	25%	15%	53	

## Discussion

This protocol is designed to gather comprehensive data on multi-level factors associated with the implementation of SBIRT in HIV care settings throughout South Africa. Building upon the scaffolding and collaborative networks established by the South Africa International TTC, the proposed study will enable us to first codify and then evaluate an SBIRT train-the-trainer cascade model on implementation outcomes at multiple levels—trainer-level (i.e., knowledge, fidelity), provider-level (i.e., attitudes, confidence, acceptability), and patient encounter-level (i.e., reach of SBIRT to eligible patients).

It will be important in carrying out this protocol that operations are able to be readily adapted across a broad range of clinical and community care environments for individuals dually at risk for HIV and problem alcohol use, and as challenges arise. For example, this protocol will require work across a range of environments for training and intervention delivery, and there will likely be differences across organizations and units within organizations in familiarity with and access to technology. Further, as the COVID-19 pandemic continues to evolve, there will likely be need for more remote rather than in-person interactions as initially planned. Accordingly, the South Africa International TTC will develop the master training manual for both face-to-face and virtual delivery to ensure maximum flexibility. Any other adjustments made in response to the COVID-19 pandemic will be documented on an ongoing basis.

Findings from the research described in this protocol will be subject to important limitations. First, due to the lack of an integrated medical record system for consistent tracking both within and across our partner organizations, we will not be able to obtain true patient-level data. Instead, we rely on patient encounter-level data captured in real time using case report forms. The real-time nature of data collection will bolster confidence in data accuracy, but the data collected will only be as strong as the data entered, and may be subject to limitations as a result of both organization and provider adherence with the protocols. Second, while we are striving to work across a wide range of clinical and community care environments, and in a wide range of geographic locations, our data may not be generalizable beyond our partner organizations.

Despite these limitations, this protocol has a number of strengths and innovations. The long-term goal of this program of research is to maximize advances in integrated HIV-alcohol treatment and care, deriving lessons in implementation that can extend to other regions confronting dual epidemics. The cascade train-the-trainer model will enable HIV service organizations to build their capacity to roll out an evidence-based intervention, SBIRT, to promote integrated HIV and alcohol treatment and care. The train-the-trainer curriculum (e.g., SBIRT Training Manual, Provider Resource Guide, fidelity scoring system, and ongoing consultation calls) aims to enhance skill in providing SBIRT training and enhance lay provider professional development and confidence in SBIRT delivery. The rollout of this curriculum will facilitate the scale-up of SBIRT across high priority regions in South Africa, therefore potentially providing benefits to patients such as enhanced screening, brief intervention, and improved referral to treatment to reduce risky drinking and improve integrated HIV-alcohol care. If the current protocol demonstrates the effectiveness of the train-the-trainer model, it will offer a reproducible implementation methodology that may be widely applied across South Africa to enhance integrated HIV and alcohol use disorder treatment. Lessons learned will be relevant for informing the scalability of evidence-based interventions in other low-and-middle income countries and other low-resource settings. Overall, this program of research will promote the efficient leveraging of scarce resources to advance implementation science around integrated HIV and alcohol treatment and care.

## Data Availability

This study has not yet started recruiting. Once data collection begins, the datasets generated during the study will be submitted to the National Institute on Alcohol Abuse and Alcoholism Data Archive (NIAAA_DA_). The NIAAA_DA_ (https://nda.nih.gov/niaaa) began accepting data in 2019 and anticipates making data publicly available in 2023. In the interim, datasets will also be available from the corresponding author on reasonable request.

## Abbreviations

AIDS: Acquired Immunodeficiency Syndrome
AUDIT: Alcohol Use Disorders Identification Test
HIV: Human immunodeficiency virus
PEPFAR: President’s Emergency Plan for AIDS Relief
SBIRT: Screening, Brief Intervention, and Referral to Treatment
TTC: Technology Transfer Center

## Footnotes

1 As of September 2020, National Advisory Board includes representatives from the National and Provincial Departments of Health (including Directorates of Mental Health and Substance Abuse, and Directorates of Drug-Resistant TB, Tuberculosis, and HIV); National and Provincial Departments of Social Development; the South Africa Central Drug Authority; the South African Medical Research Council; the South African National Council on Alcoholism and Drug Dependence; the South African National AIDS Council; the South African Federation for Mental Health; and HIV treatment, care, and advocacy organizations (TB/HIV Care, ANOVA Health Institute, Lifeline, and Médecins Sans Frontières).

## Declarations

### Clinical trial registration

This study protocol does not meet the definition of a clinical trial per the National Institute on Health (NIH) Guidelines. This protocol develops and evaluates a train-the-trainer model: the only human subjects are trainers and providers. The trainer-level outcomes are indicators of trainer knowledge and fidelity, whereas the provider-level outcomes are measures of confidence, attitudes, and acceptability. Patient encounters are tracked but patients do not participate as human subjects and no patient health outcomes are collected. The research participants do not provide any data on health-related outcomes or behaviors.
